# Pulmonary thromboembolism in hospitalised COVID-19 patients at moderate to high risk by Wells score: a report from Lombardy, Italy

**DOI:** 10.1259/bjr.20200407

**Published:** 2020-07-31

**Authors:** Lorenzo Monfardini, Mauro Morassi, Paolo Botti, Roberto Stellini, Luca Bettari, Stefania Pezzotti, Marco Alì, Cristian Giuseppe Monaco, Veronica Magni, Andrea Cozzi, Simone Schiaffino, Claudio Bnà

**Affiliations:** 1 Department of Radiology, Fondazione Poliambulanza Istituto Ospedaliero, Via Leonida Bissolati 57, Brescia, Italy; 2 Infectious Diseases Service, Fondazione Poliambulanza Istituto Ospedaliero, Via Leonida Bissolati 57, Brescia, Italy; 3 Cardiology Unit, Cardiovascular Department, Fondazione Poliambulanza Istituto Ospedaliero, Via Leonida Bissolati 57, Brescia, Italy; 4 Unit of Radiology, IRCCS Policlinico San Donato, Via Rodolfo Morandi 30, San Donato Milanese, Italy; 5 Unit of Diagnostic Imaging and Stereotactic Radiosurgery, C.D.I. Centro Diagnostico Italiano S.p.A., Via Simone Saint Bon 20, Milano, Italy; 6 Medical School, Università degli Studi di Milano, Via Festa del Perdono 7, Milano, Italy; 7 Department of Biomedical Sciences for Health, Università degli Studi di Milano, Via Luigi Mangiagalli 31, Milano, Italy

## Abstract

**Objectives:**

To present a single-centre experience on CT pulmonary angiography (CTPA) for the assessment of hospitalised COVID-19 patients with moderate-to-high risk of pulmonary thromboembolism (PTE).

**Methods:**

We analysed consecutive COVID-19 patients (RT-PCR confirmed) undergoing CTPA in March 2020 for PTE clinical suspicion. Clinical data were retrieved. Two experienced radiologists reviewed CTPAs to assess pulmonary parenchyma and vascular findings.

**Results:**

Among 34 patients who underwent CTPA, 26 had PTE (76%, 20 males, median age 61 years, interquartile range 54–70), 20/26 (77%) with comorbidities (mainly hypertension, 44%), and 8 (31%) subsequently dying. Eight PTE patients were under thromboprophylaxis with low-molecular-weight heparin, four PTE patients had lower-limbs deep vein thrombosis at ultrasound examination (performed in 33/34 patients). Bilateral PTE characterised 19/26 cases, with main branches involved in 10/26 cases. Twelve patients had a parenchymal involvement >75%, the predominant pneumonia pattern being consolidation in 10/26 patients, ground glass opacities in 9/26, crazy paving in 5/26, and both ground glass opacities and consolidation in 2/26.

**Conclusion:**

COVID-19 patients are prone to PTE.

**Advances in knowledge:**

PTE, potentially attributable to an underlying thrombophilic status, may be more frequent than expected in COVID-19 patients. Extension of prophylaxis and adaptation of diagnostic criteria should be considered.

## Introduction

The ongoing novel coronavirus disease 2019 (COVID-19) pandemic has been severely disrupting the workflow of health-care systems even in areas with adequately funded medical networks. In Lombardy, the most affected region in Italy, intensive care units (ICUs) are put under deep pressure by the high percentage of critically ill COVID-19 patients,^1^ a figure reported to hover around 14% of all hospitalised patients.^2^

COVID-19 is more and more recognised as a systemic disease^3^: from a cardiovascular point of view, the already postulated relationship between infection by seasonal respiratory viruses and a pro-thrombotic status^4^ has also been observed for patients infected with severe acute respiratory syndrome coronavirus 2 (SARS-CoV-2).^5–7^ Cases of pulmonary thromboembolism in these patients have being increasingly described^8,9^ and observed in areas affected by the SARS-CoV-2 pandemic, their monitoring being advised also by clinical guidance and position papers.^7,10^ From 21 February 2020, when the first Italian cases of COVID-19 were reported in Lombardy,^1^ our Radiology department situated in Brescia – one of the worst hit areas of this region – has also being seeing an ever-rising number of COVID-19 patients with acute pulmonary embolism. The aim of this paper is therefore to present our single-centre experience about clinical and CT features of pulmonary thromboembolism in COVID-19 patients.

## Methods and materials

### Study population

Approval for this retrospective monocentric observational study was obtained from the competent Ethics Committee (Comitato Etico di Brescia). Consecutive patients hospitalised at Fondazione Poliambulanza Istitututo Ospedaliero (Brescia) with SARS-CoV-2 infection confirmed by reverse transcriptase-polymerase chain reaction (RT-PCR, Novel Coronavirus PCR Fluorescence Diagnostic Kit, BioGerm Medical Biotechnology, Shanghai, China) from 1 March to 31 March 2020 were considered eligible to be included in this study. If a patient’s clinical conditions gave rise to a suspicion of pulmonary thromboembolism, chest CT was performed with the administration of iodinated contrast agents. Our institutional database was reviewed and COVID-19 patients undergoing CT pulmonary angiography (CTPA) for clinical suspicion of acute pulmonary thromboembolism were selected.

Complete clinical data were retrievable only for patients in which CTPA-detected pulmonary thromboembolism occurred between hospital admission and discharge, their medical records being then reviewed to extract the following parameters: demographics, comorbidities, symptoms at acceptance, lower limbs venous ultrasound doppler, time between admission and pulmonary thromboembolism diagnosis, need of ICU treatment after SARS-CoV-2 infection, and patients’ outcome (discharge or death).

### Procedures

Pulmonary thromboembolism suspicion was defined as moderate or high according to clinical findings incorporated into the Wells Score^11^: patients with moderate risk and positive D-dimer and those with high risk (regardless of D-dimer values) were referred for CTPA. Bedside lower limbs venous doppler examination was performed by a vascular surgeon, in a maximum timeframe of 24 hours before CTPA, to all patients with suspected pulmonary thromboembolism.

CT scans were performed on a 16-slice CT scanner (LightSpeed RT 16, General Electric Healthcare, Chalfont St. Giles, UK) before and after intravenous injection of a 1 mL/kg of body weight dose of a iodinated contrast agent (Iopamidol 370 mg/dL). Scanning parameters included: 1.25 mm section thickness, 1.25 mm interval, 120 kVp, bolus track technique with region of interest placed in the main pulmonary artery trunk.

### Image review

CT images were independently reviewed by two experienced thoracic radiologists (MM and PB) with 15 and 20 years of experience, respectively. Discordances in evaluation were solved by consensus. They assessed both lung parenchymal and vascular involvement, being blinded for clinical data. All images were examined on a dedicated CTPA window (width, 700 HU; level, 80 HU), and on both lung (width, 1500 HU; level, −700 HU) and mediastinal (width, 350 HU; level, 40 HU) windows.

Lung parenchymal involvement was categorised according to the predominant CT feature (presence of ground-glass opacities, consolidation, crazy paving), its extent being visually assessed by adapting the method proposed by Bernheim et al,^12^ as follows: 0% (absent); 1–25% (minimal); 26–50% (mild); 51–75% (moderate); over 75% (severe).

To assess vascular involvement, the presence of pulmonary thromboembolism in both lungs was classified considering principal, lobar, segmental, subsegmental, and peripheral arterial involvement. Pulmonary artery maximum diameter was recorded on axial images at bifurcation and classified as normal or increased according to the following cut-off values: >30 mm for males and >29 mm for females.^13^ Presence of pleural and pericardial effusion was also recorded, along with the ratio between the right and left ventricular diameters.

### Statistical analysis

Continuous data were reported as median and interquartile range (IQR). In view of the limited sample size, data were analysed for descriptive purposes only.

## Results

In the study period, 34 out of 1,207 confirmed COVID-19 patients admitted to our institution (Fondazione Poliambulanza Istituto Ospedaliero, Brescia) underwent CTPA because of sudden oxygen desaturation coupled with a moderate to high risk of pulmonary thromboembolism according to the Wells Score and D-dimer values, 26/34 (76%) being positive for pulmonary thromboembolism, and 8/34 (24%) negative. All patients, excluding the one deceased shortly after Emergency Department admission, were investigated with doppler ultrasound the same day or the day before CTPA, only 4/34 (12%) presenting deep vein thrombosis (data retrieved by electronic report, without images revision), all of them with a subsequent CTPA finding of pulmonary thromboembolism.

The 26 patients with pulmonary thromboembolism had a median age of 61 years (IQR 54–70), 20 of them being males (77%). A female patient died 6 hours after admission to our Emergency Department, without any available medical history. Patients presented with comorbidities in 84% of cases (21/25), the most frequent being arterial hypertension in 44% (11/25), type II diabetes in 16% (4/25), oncological history in 12% (3/25) and obesity in 8% (2/25). One patient had coronary artery disease and surgical treatment to the ascending aorta 16 years before, while another was tetraplegic after suffering a spinal cord injury 13 years before. One of the two obese patients was taking hormonal therapy. One patient had trisomy 12 with an history of hepatitis C and lymphoblastic leukaemia (Table 1).

**Table 1.** Demographic, comorbidities and outcomes of the 26 patients with CT angiographic signs of pulmonary thromboembolism

Sex	Age	Comorbidities	Days between admittance and PTE	Anti coagulant therapy before PTE	DVT	D-dimer (ng/ml)	ICU admission	Clinical outcome
M	69	Prostate hypertrophy	8	LMWH	No	>20,000	Yes	Discharged
M	61		3	LMWH	No	>20,000	Yes	Discharged
F	62		0	No	Yes	N/A ^a^	No	Discharged
M	59	Hypertension	6	No	No	>20,000	No	Discharged
M	59	Atrial fibrillation	7	No	No	14,101	Yes	Dead
M	53	Hypertension and DM2	10	No	Yes	N/A ^a^	Yes	Dead
F	47		4	LMWH	No	>20,000	Yes	Discharged
M	53	Obesity	7	LMWH	No	3,886	Yes	Discharged
M	56		12	LMWH	No	8,441	No	Discharged
M	73	Hypertension, DM2, and malignancy	0	No	Yes	N/A ^a^	No	Discharged
M	70	Hypertension	12	No	Yes	N/A ^a^	No	Discharged
F	71	Hypertension and DM2	19	No	No	12,006	No	Discharged
F	52	^b^	0	^b^	^b^	N/A ^a^	No	Dead
F	43	Obesity	0	No	No	>20,000	No	Discharged
M	71	Hypertension and previous MI	8	No ^c^	No	N/A ^a^	No	Dead
M	50	Crohn's disease	1	No	No	2,593	No	Discharged
M	58	Malignancy	1	LMWH	No	4,386	No	Discharged
M	54	Post-traumatic tetraplegia	6	LMWH	No	11,271	No	Dead
M	65	Hypertension	0	No	Yes	N/A ^a^	Yes	Discharged
M	54	Asthma	11	No	No	>20,000	Yes	Dead
F	60	Malignancy and HCV chronic infection	13	No	No	>20,000	No	Dead
F	69	Hypertension and DM2	5	LMWH	No	N/A ^a^	No	Dead
M	71	Hypertension	13	No	No	9,774	No	Dead
M	73		16	No	No	N/A ^a^	Yes	Dead
M	68	Hypertension	2	No	No	>20,000	No	Discharged
M	63	Hypertension	30	No	No	N/A ^a^	No	Discharged

DM2, diabetes mellitus type 2; DVT, deep venous thrombosis; ICU, intensive care unit; LMWH, low-molecular-weight heparin; MI, myocardial infarction; PTE, pulmonary thromboembolism.

a Due to sudden unexplained worsening of clinical conditions and high risk of pulmonary thromboembolism according to the Wells Score, CTPA was performed before D-dimer levels could be obtained.

b Data not available, this patient died 6 hours after Emergency Department acceptance without any available medical history.

c The patient reported taking antiplatelet drug therapy (acetylsalicylic acid)

At acceptance, fever (82%), dyspnoea (78%) and cough (47%) were the most prevalent clinical manifestations. Fever had appeared from 5 to 14 (mean 8.7) days before Emergency Department presentation, associated with cough and/or followed by dyspnoea. Ten patients (38%) ultimately needed ICU treatment.

Median time distance between acceptance and CTPA-detected pulmonary thromboembolism was 7 days (IQR 7–12). Among 26 patients with pulmonary thromboembolism, in 5/26 cases (19%), CT scan was performed in the Emergency Department before ward admission, in 6/26 (23%) during ICU stay and in the remaining 15/26 (58%) during medical ward hospitalisation. During hospitalisation, 8 of the 26 patients (31%) with subsequent signs of pulmonary thromboembolism at CTPA were receiving low molecular weight heparin at prophylactic dosage.

At least 2 weeks of follow-up were available for all patients with pulmonary thromboembolism and, at the end of the study period, 16/26 (62%) patients had been discharged from hospital while 10/26 (38%) had died.

### CTPA findings

In the 26 patients with pulmonary thromboembolism, overall lung parenchyma involvement was less than 25% in 1 patient (4%), between 26 and 50% in 2/26 (8%) patients, between 51 and 75% in 11/26 (42%) patients, and higher than 75% in 12/26 (46%) patients. Ground glass opacities were the predominant pattern in 9/26 patients (35%), consolidations in 10/26 (38%), ground glass and consolidations in 2/26 (8%), and crazy paving in 5/26 (19%). Of note, in 17/26 patients (65%) all three patterns variously coexisted throughout the lungs (Table 2).

**Table 2.** Chest involvement data of patients with CT angiographic signs of pulmonary thromboembolism, including involvement of pulmonary arterial branches, pleural and pericardial effusion and lung parenchymal involvement, expressed in percentage

Patient	Right lobes involved	Left lobes involved	Main branches involvement	Pleural effusion	Pericardial effusion	Lung parenchyma involvement (%)
**1**	3 (U-M-L)	-	No	No	No	>75
**2**	2 (U-L)	-	No	No	No	>75
**3**	3 (U-M-L)	2 (U-L)	Yes	No	No	51–75
**4**	-	2 (U-L)	Yes	Bilateral	No	51–75
**5**	3 (U-M-L)	2 (U-L)	No	Bilateral	Yes	>75
**6**	2 (U-L)	-	No	Bilateral	No	>75
**7**	2 (U-L)	2 (U-L)	Yes	No	No	>75
**8**	3 (U-M-L)	2 (U-L)	Yes	Bilateral	No	51–75
**9**	3 (U-M-L)	2 (U-L)	Yes	No	No	51–75
**10**	3 (U-M-L)	2 (U-L)	Yes	No	No	51–75
**11**	3 (U-M-L)	2 (U-L)	Yes	No	No	>75
**12**	3 (U-M-L)	1 (U)	No	No	No	>75
**13**	1 (L)	1 (U)	No	Unilateral	No	>75
**14**	3 (U-M-L)	2 (U-L)	Yes	No	No	26–50
**15**	1 (U)	1 (L)	No	No	No	51–75
**16**	-	2 (U-L)	No	Unilateral	No	26–50
**17**	1 (L)	1 (L)	No	No	No	51–75
**18**	-	1 (L)	No	Bilateral	Yes	>75
**19**	3 (U-M-L)	2 (U-L)	Yes	Unilateral	No	<25
**20**	2 (M-L)	1 (L)	No	No	No	51–75
**21**	1 (L)	-	No	Bilateral	No	>75
**22**	2 (U-L)	2 (U-L)	Yes	Unilateral	No	>75
**23**	3 (U-M-L)	2 (U-L)	No	Bilateral	No	51–75
**24**	1 (L)	-	No	No	No	>75
**25**	2 (U-L)	2 (U-L)	No	No	No	51–75
**26**	2 (U-L)	-	No	Bilateral	Yes	51–75

U, upper lobe; M, middle lobe; L, lower lobe.

**Figure 1.**
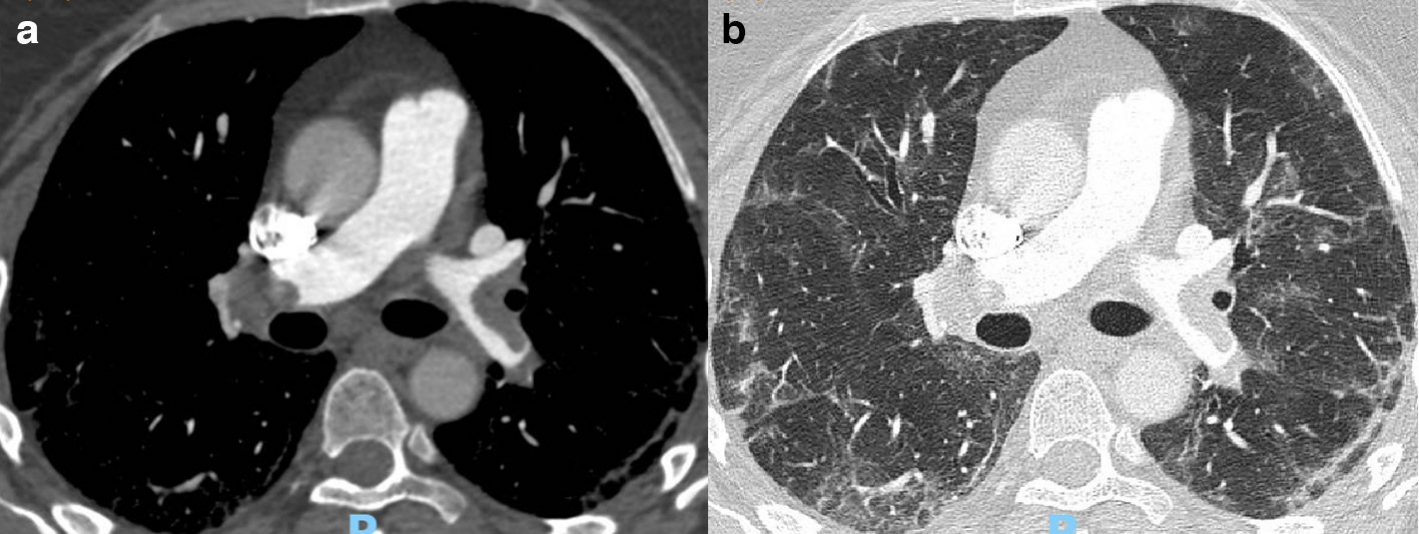
CT pulmonary angiography performed at Emergency Department acceptance in a 62-year-old female with bilateral pulmonary thromboembolism involving lobar, segmental and subsegmental arterial branches. She was subsequently hospitalised, treated with heparin therapy, and discharged after 21 days of hospitalisation, without intensive care need. In (A), the mediastinal setting image shows bilateral pulmonary thromboembolism. In (B), the lung setting shows bilateral ground-glass parenchymal and interstitial involvement, mainly in the posterior segments.

All patients with pulmonary thromboembolism presented multiple thromboembolic localisations, with bilateral findings (Figure 1) in 19/26 cases (73%), right lung only findings in 6/26 (23%), and left lung only in 1 patient (4%). Main branches were involved in 10/26 cases (38%), 8 of them with bilateral involvement. Lobar involvement was noted in 5/26 patients (19%), more frequently in the lower lobes (4 out of 5 cases). Segmental and subsegmental pulmonary thromboembolism occurred in the remaining 11/26 (42%) patients, of whom 5 with findings in both upper and lower lobes, 5 in the upper or lower lobe, and 1 in upper lower and middle lobe. No patient presented pulmonary infarcts. Pulmonary artery diameter was increased in 12 out of 26 patients (46%), with a median value of 30 mm (IQR 27–31 mm). 12 patients (46%) had an increased right-to-left ventricular ratio, for a median value of 1 (IQR 0.93–1.40). Overall, eight patients had bilateral pleural effusion and four unilateral. Only two patients had pericardial effusion.

## Discussion

Increasing evidence is demonstrating how COVID-19, whose principal manifestation appears to be pneumonia, is actually a systemic disease.^3,7^ The association between inflammation and thrombosis has been reported in several conditions,^4^ and the systemic inflammatory state induced by COVID-19 is no exception.^4–6^ In these patients, direct pulmonary endothelial damage may contribute to the inflammation-induced thrombotic profile,^6,14^ with further increase in local thrombosis risk in the lung.^10^

In our case series of patients with moderate-to-high pre-test probability of pulmonary thromboembolism, 76% showed signs of pulmonary thromboembolism at CTPA, that was however associated with ultrasound-detected lower-limbs deep vein thrombosis only in four cases (15%). Moreover, considering the actual prevalence of pulmonary thromboembolism in our small cohort of COVID-19 patients with moderate to high risk according to the Wells Score and D-dimer values, we found a two- to fivefold increase to the prevalence reported by the original study by Wells et al.^11^

These data support the hypothesis that sees COVID-19 patients having an increased thromboembolic risk that tends to manifest itself as pulmonary arterial thrombosis and non-pulmonary thromboembolism even in patients under thromboembolic prophylaxis with anticoagulant therapy, in the association of global inflammation-induced thrombophilia and direct pulmonary vascular damage.^7,15^ This scenario also possibly indicates that the sensitivity of conventional diagnostic criteria could be insufficient to correctly diagnose pulmonary thromboembolism in COVID-19 patients.^10^

Limitations of this work include its monocentric and cross-sectional nature: the quite short period elapsed from the outbreak of COVID-19 in our area and the still-ongoing emergency hindered the possibility to obtain full clinical and anamnestic data, long-term prognostic information, as well as to fully exclude a selection bias linked to the eventuality that some patients complying our inclusion criteria were not referred for CTPA, because they had contraindications to the administration of iodinated contrast agents or because CT examinations were temporarily unavailable due to the unprecedented number of patients needing unenhanced chest CT for triaging purposes. Further data regarding pulmonary arterial thrombosis and non-pulmonary thromboembolism, along with a clearer understanding of the pathogenesis of these phenomena in COVID-19 patients, are therefore much needed to guide and refine the clinical management of hospitalised COVID-19 patients. In particular, it is paramount to ascertain the burden of the increased thromboembolic risk in patients who already exhibit a globally critical clinical profile, to correctly balance the need for thromboembolic prophylaxis and each patient’s risk of bleeding.^10^

## Conclusion

COVID-19 patients seem to be prone to thromboembolic pulmonary events of still undefined characterisation in their arterial, venous, or even combined origin. Since these events are potentially triggered by an underlying thrombophilic state, prophylactic measures should be carefully tailored, also considering adaptations to diagnostic criteria and pathways.
